# Scanning electron microscopy of panitumumab-induced eyelash and hair alterations – Pili canaliculi^☆^

**DOI:** 10.1016/j.abd.2021.03.011

**Published:** 2022-01-15

**Authors:** Debora Sarzi Sartori, Antônia Larangeira de Almeida, Gabriel Santana Pereira de Oliveira, Hiram Larangeira de Almeida Jr

**Affiliations:** a Post-graduation Program in Health, Universidade Católica de Pelotas, Pelotas, RS, Brazil; b Department of Dermatology, Universidade Federal de Pelotas, Pelotas, RS, Brazil; c Dermatology League, Universidade Federal de Pelotas, Pelotas, RS, Brazil

**Keywords:** Microscopy, electron, Panitumumab, Scalp dermatoses

## Abstract

Panitumumab is a monoclonal antibody against the epidermal growth factor receptor used in metastatic colorectal cancer; in addition to tumor cells, it acts on epidermal keratinocytes and on the outer root sheath and presents skin toxicity in up to 90% of cases. A scanning electron microscope was used to examine the eyelashes and hairs of a 65-year-old patient with eyelash trichomegaly, curly hair, and paronychia undergoing treatment with panitumumab. Grooving in the hair shafts were identified, which were more evident in the eyelashes. Similar to oral epidermal growth factor inhibitors (erlotinib and gefitinib), panitumumab can cause acquired pili canaliculi.

Epithelial growth factor (EGF) inhibitors are considered essential for the treatment of several epithelial tumors and are part of the list of target therapies, as they act on a specific signaling pathway and therefore have a better adverse effects profile when compared to conventional chemotherapy.^1^ They can be small molecules for oral use, acting intracellularly, such as erlotinib and gefitinib, and also immunobiologicals targeting the receptor itself (EGFR), such as panitumumab and cetuximab. Among the skin adverse effects papulopustular eruptions are the most frequent,^2^, ^3^ in addition to pruritus, xerosis, periungual pyogenic granuloma, eyelash trichomegaly, hair thickening, and curling.^4^, ^5^

Panitumumab is a fully human anti-EGFR monoclonal antibody used primarily to treat metastatic colorectal cancer. However, in addition to its action on tumor cells, it also acts on other tissues that have EGFR expression, particularly on the keratinocytes in the basal layer of the epidermis and in the outer sheath of hair follicles, leading to skin toxicity in up to 90% of cases.^6^

Although there have been reports describing the ultrastructural aspects of hair secondary to oral EGFR inhibitors, such as gefitinib^7^ and erlotinib,^8^ there are no reports of electron microscopy analysis of these effects on hair shafts with monoclonal antibodies that inhibit this receptor.

## Case report

This case report describes a 65-year-old male patient with a malignant colorectal neoplasm. Surgical treatment with hemicolectomy and adjuvant chemotherapy was performed. A year later, he started another chemotherapy regimen due to the appearance of liver metastases; however, this treatment was not effective, so treatment with panitumumab was initiated. After six months of treatment, the patient showed pyogenic periungual granulomas and eyelash alterations, which became elongated and straightened, requiring cutting for aesthetic reasons, his hair became slightly curly and rougher to the touch. Eyelash and hair fragments were collected by cutting them with scissors for *in natura* analysis using scanning electron microscopy.

**Figure 1:**
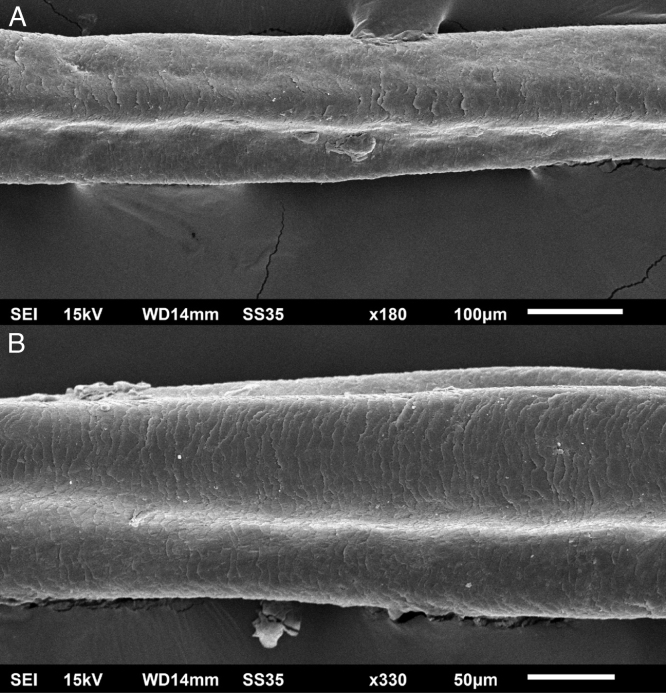
Scanning electron microscopy - Eyelash examination - small (A) and medium magnification (B), showing grooves in the hair shaft (×180, ×330).

**Figure 2:**
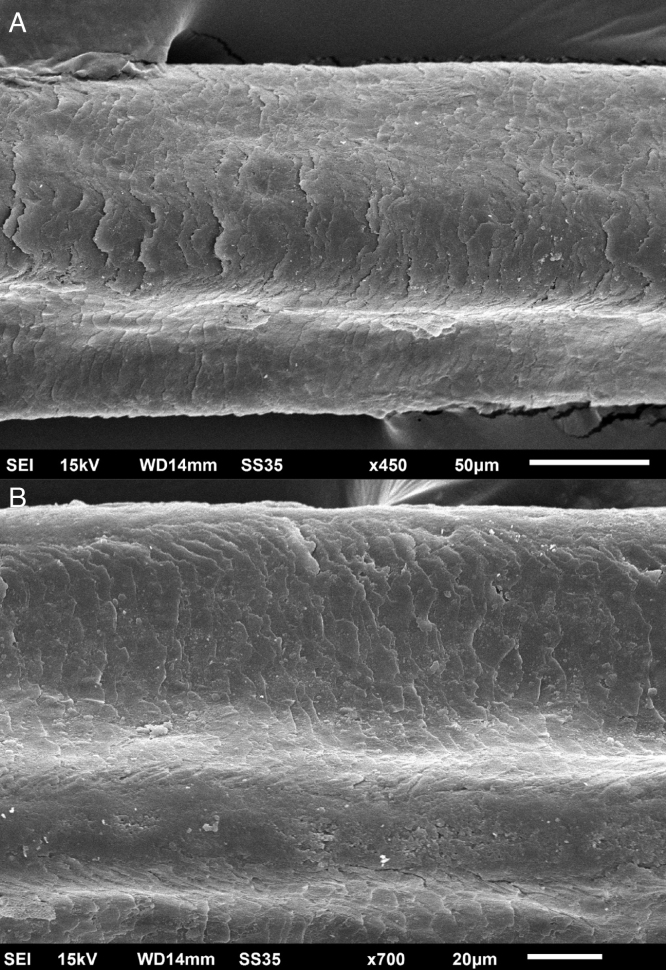
Scanning Electron Microscopy – Eyelash examination – (A), high magnification, showing grooving in the hair shaft and cuticle irregularity (×450). (B), High magnification, showing two grooves in the hair shaft – arrows (×700).

**Figure 3:**
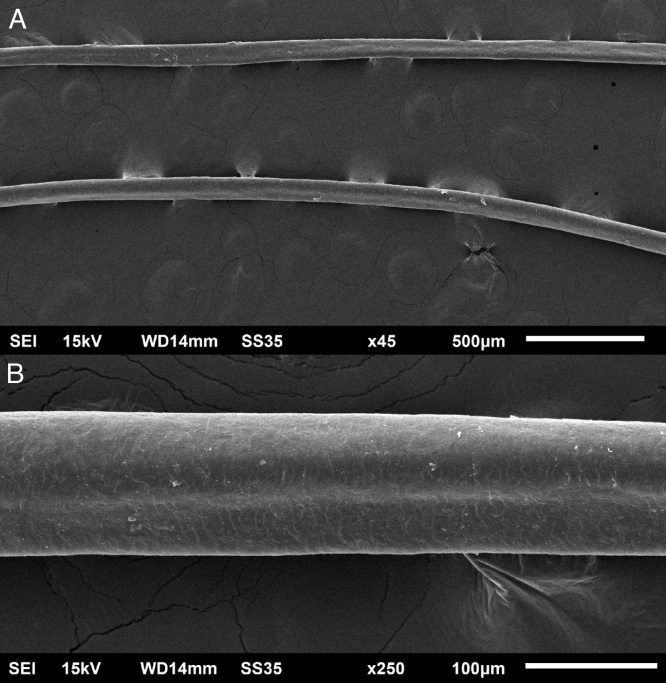
Scanning electron microscopy - Hair examination - small (A) and medium magnification (B), showing grooves in the hair shaft (×45, ×250).

**Figure 4:**
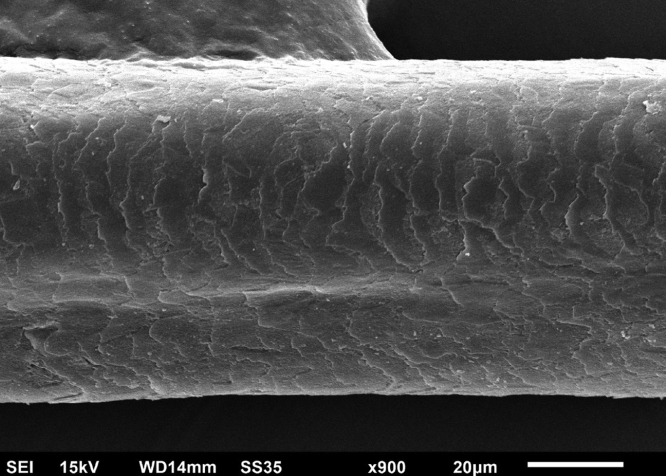
Scanning electron microscopy - Hair examination - large magnification showing slight cuticle disorganization (×900).

The eyelash examination showed well-developed grooves at medium magnifications (Fig. 1), high magnifications showed cuticle disorganization, in addition to the grooving, and some hair shafts had more than one groove (Fig. 2). Examination of head hairs also revealed grooves (Fig. 3) at small and medium magnifications, less evident than in the eyelashes. High magnifications showed slight cuticle disorganization (Fig. 4).

## Discussion

The ultrastructural findings observed secondary to the use of panitumumab are similar to those previously described for erlotinib and gefitinib, in which grooving was described and classified as pili canaliculi. In the case of erlotinib, eyelash trichomegaly and curly hairs, were seen, ultrastructurally grooves and hair twisting were observed. In the reported case of gefitinib use, the alterations were subclinical with discrete grooving. The case investigated here had important eyelash trichomegaly, with more discreet alterations of head hairs, both clinically and on ultrastructural examination. Panitumumab prolongs the anagen phase, hence the occurrence of trichomegaly, with more evident effects on the eyelashes.

These findings demonstrate that the ultrastructural aspects of hair follicle changes by EGF inhibition are independent of the inhibition pathway. Both intracellular tyrosine kinase inhibition by oral small molecules and competitive inhibition of the EGFR binding site on the cell surface with monoclonal antibodies, produce similar ultrastructural alterations.

## Financial support

None declared.

## Authors' contributions

Debora Sarzi Sartori: Approval of the final version of the manuscript; design and planning of the study; drafting and editing of the manuscript; collection, analysis, and interpretation of data; intellectual participation in the propaedeutic and/or therapeutic conduct of the studied cases; critical review of the literature; critical review of the manuscript.

Antônia Larangeira de Almeida: Approval of the final version of the manuscript; drafting and editing of the manuscript; intellectual participation in the propaedeutic and/or therapeutic conduct of the studied cases; critical review of the literature; critical review of the manuscript.

Gabriel Santana Pereira de Oliveira: Approval of the final version of the manuscript; drafting and editing of the manuscript; intellectual participation in the propaedeutic and/or therapeutic conduct of the studied cases; critical review of the literature; critical review of the manuscript.

Hiram de Almeida Jr: Approval of the final version of the manuscript; design and planning of the study; drafting and editing of the manuscript; collection, analysis, and interpretation of data; effective participation in research orientation; intellectual participation in the propaedeutic and/or therapeutic conduct of the studied cases; critical review of the literature; critical review of the manuscript.

## Conflicts of interest

None declared.
